# Description of new species of *Mycobacterium terrae* complex isolated from sewage at the São Paulo zoological park foundation in Brazil

**DOI:** 10.3389/fmicb.2024.1335985

**Published:** 2024-01-23

**Authors:** Camila Lopes Romagnoli, Emilyn Costa Conceição, Edson Machado, Leonardo Bruno Paz Ferreira Barreto, Abhinav Sharma, Natalia Maria Silva, Lucas Evangelista Marques, Maria Aparecida Juliano, Maria Cristina da Silva Lourenço, Luciano Antonio Digiampietri, Philip Noel Suffys, Sylvia Cardoso Leão, Cristina Viana-Niero

**Affiliations:** ^1^Departamento de Microbiologia, Imunologia e Parasitologia, Universidade Federal de São Paulo, São Paulo, Brazil; ^2^Laboratório de Bacteriologia e Bioensaios em Micobactérias, Instituto Nacional de Infectologia Evandro Chagas, Fundação Oswaldo Cruz, Rio de Janeiro, Brazil; ^3^DSI-NRF Centre of Excellence for Biomedical Tuberculosis Research, SAMRC Centre for Tuberculosis Research, Division of Molecular Biology and Human Genetics, Faculty of Medicine and Health Sciences, Stellenbosch University, Cape Town, South Africa; ^4^Laboratório de Biologia Molecular Aplicada a Micobactérias, Instituto Oswaldo Cruz, Fundação Oswaldo Cruz, Rio de Janeiro, Brazil; ^5^Departamento de Biofísica, Universidade Federal de São Paulo, São Paulo, Brazil; ^6^Escola de Artes, Ciências e Humanidades, Universidade Federal de São Paulo, São Paulo, Brazil

**Keywords:** *Mycolicibacter*, taxonomy, non-tuberculous mycobacteria, whole-genome sequencing, MALDI-TOF-MS, *Mycobacterium terrae* complex, Brazil

## Abstract

Five mycobacterial isolates from sewage were classified as members of the genus *Mycobacterium* but presented inconclusive species assignments. Thus, the isolates (MYC017, MYC098, MYC101, MYC123 and MYC340) were analyzed by phenotypical, biochemical, matrix-assisted laser desorption/ionization time-of-flight mass spectrometry (MALDI-TOF-MS) and genomic features to clarify their taxonomic position. Phenotypic analysis and biochemical tests did not distinguish these isolates from other non-pigmented mycobacteria. In contrast, MALDI-TOF MS analysis showed that isolates were not related to any previously described *Mycobacterium* species. Comparative genomic analysis showed values of ANI and dDDH between 81.59–85.56% and 24.4–28.8%, respectively, when compared to the genomes of species of this genus. In addition, two (MYC101 and MYC123) presented indistinguishable protein spectra from each other and values of ANI = 98.57% and dDDH = 97.3%, therefore being considered as belonging to the same species. Phylogenetic analysis grouped the five isolates within the *Mycobacterium terrae* complex (MTC) but in a specific subclade and separated from the species already described and supported by 100% bootstrap value, confirming that they are part of this complex but different from earlier described species. According to these data, we propose the description of four new species belonging to the *Mycobacterium* genus: (i) *Mycobacterium defluvii* sp. nov. strain MYC017*^T^* (= ATCC TSD-296*^T^* = JCM 35364*^T^*), (ii) *Mycobacterium crassicus* sp. nov. strain MYC098*^T^* (= ATCC TSD-297*^T^* = JCM 35365T), (iii) *Mycobacterium zoologicum* sp. nov. strain MYC101*^T^* (= ATCC TSD-298*^T^* = JCM 35366*^T^*) and MYC123 (= ATCC BAA-3216 = JCM 35367); and (iv) *Mycobacterium nativiensis* sp. nov. strain MYC340*^T^* (= ATCC TSD-299*^T^* = JCM 35368*^T^*).

## 1 Introduction

Non-tuberculous mycobacteria (NTM) are species belonging to the genus *Mycobacterium* unrelated to the *Mycobacterium tuberculosis* complex and *Mycobacterium leprae*. Some NTM have similar characteristics and are grouped in complexes. The *Mycobacterium terrae* complex (MTC) was defined in 1981 by the International Working Group in Mycobacterial Taxonomy (IWGMT) and comprised two species, *Mycobacterium terrae* and *Mycobacterium non-chromogenicum*, which are non-pigmented and have intermediate growth rate (requiring 5–15 days to form visible colonies in solid media but phenotypic tests cannot clearly differentiate them (Tortoli, 2003; Tortoli et al., 2013). In the 1990s, a unique genetic signature, a two-nucleotide insertion in helix 18 of the 16S rRNA gene, was described as the most reliable marker for the attribution of mycobacterial species to the MTC (Kirschner et al., 1993; Springer et al., 1996; Tortoli et al., 2013).

Today, the MTC comprises 16 species, 13 of which were validly published,^1^ are ubiquitous and considered as potentially opportunistic pathogens. They have been isolated from the environment (water and soil), clinical specimens and animals. Most species are recognized as environmental contaminants of sputum but can also cause pulmonary infections, and tenosynovitis and osteomyelitis, arising from direct inoculation injuries (Vasireddy et al., 2016, 2017; Abudaff and Beam, 2018). *Mycobacterium hiberniae*, “*Mycobacterium icosiumassiliensis*” and *Mycobacterium minnesotensis* have not yet been documented as responsible for human infections (Hannigan et al., 2013; Makovcova et al., 2015; Dibaj et al., 2020).

In a previous study, we assessed a large diversity of bacteria belonging to the *Mycobacteriaceae* family isolated from different sources of water samples in a zoo in the state of São Paulo - Brazil, as well as a variety of potential new species (Romagnoli et al., 2020). Here, we present five of these isolates as candidates for being new species belonging to MTC, after being characterized by biochemical and cultural tests, matrix-assisted laser desorption/ionisation time-of-flight mass spectrometry (MALDI-TOF-MS) and genomic features. Our results show that the strains represent four new species of the *Mycobacterium* genus and contribute to the knowledge of the taxonomy of this group of bacteria.

## 2 Materials and methods

### 2.1 Bacterial strains and cultivation conditions

Strains MYC017, MYC098, MYC101, MYC123 and MYC340 were isolated from sewage at the São Paulo Zoological Park in Brazil (Romagnoli et al., 2020). Bacteria were cultivated in Middlebrook 7H10 medium supplemented with 0.5% glycerol and 10% OADC (oleic acid, albumin, dextrose, and catalase - Becton Dickinson, Franklin Lakes, USA) at 30°C for up to 10 days.

### 2.2 Morphology and phenotypic characteristics

The morphological characteristics of the colonies from the different isolates were observed during culturing in 7H10-OADC and after Ziehl-Neelsen (ZN) staining. Growth time, pigment production and temperature growth were evaluated at 30°C, 37°C and 42°C. In addition, classical biochemical tests were performed, including growth in a medium with thiophene 2-carboxylic acid hydrazide (TCH), growth in 5% NaCl, Tween 80 hydrolysis, potassium tellurite reduction, iron uptake, nitrate reduction, hydrolysis of urea, growth in MacConkey, and semiquantitative catalase (Kent and Kubica, 1985).

### 2.3 Antimicrobial susceptibility test

To perform an antimicrobial susceptibility test (AST), we used the method established by the Clinical Laboratory Standards Institute (CLSI) to determine the Minimum inhibitory concentrations (MIC) (Clinical and Laboratory Standards Institute [CLSI], 2018). From isolated colonies, we prepared a bacterial suspension with an OD of 0.5 on the McFarland scale, adjusted to the final inoculum of approximately 5 × 10^5^ CFU/mL in Muller-Hinton II cation-adjusted medium (Becton Dickinson, Franklin Lakes, USA). The suspensions were inoculated in a 96-well microplate with dilutions indexed to base 2. We tested the following drugs: amikacin, ciprofloxacin, clarithromycin, doxycycline, trimethoprim associated with sulfamethoxazole, moxifloxacin, rifampicin, isoniazid, streptomycin, and ethambutol. All tests were performed in triplicate and incubated at 30°C, and growth was observed after 3 and 7 days. Test interpretation was performed according to CLSI, document M62, which recommends for MTC evaluate the same first- and second-line drugs for *M. kansasii* using the same breakpoints (Woods et al., 2011; Clinical and Laboratory Standards Institute [CLSI], 2018) and published data (Cloud et al., 2006; Masaki et al., 2006; Mun et al., 2008; Sahraoui et al., 2011; Tortoli et al., 2013; Zhang et al., 2013; Djouadi et al., 2016; Vasireddy et al., 2016) (Supplementary Table 1).

### 2.4 DNA extraction and whole-genome sequencing

For DNA preparation, the bacterial mass was added in a tube containing 0.06 g of glass beads with 0.5 mm diameter (BioSpec, Bartlesville, USA) and 100 μL of Tris-EDTA (TE) 1X. After vigorous homogenization by vortex for 5 min, the tubes were incubated at 80°C for 10 min and centrifuged at 5000 × *g* for 2 min. Following, genomic DNA extraction was performed using the QIAamp DNA Mini Kit (Qiagen, Hilden, Germany) following the manufacturer’s recommendations (Silva et al., 2023). DNA quality was verified using Qubit^®^ dsDNA HS (High Sensitivity) Assay Kits (Thermo Fisher Scientific, Waltham, United States). The whole-genome sequencing (WGS) was carried out on a MiSeq instrument (Illumina San Diego, United States), using 2 × 150 paired-end chemistry and the Nextera XT library preparation kit (Illumina, San Diego, United States).

### 2.5 Genome features

A script developed by the authors was used to remove adaptors and primers. The read quality was verified using FastQC v0.11.9 before and after the trimming by Trimmomatic v0.35 (Bolger et al., 2014; Andrews, 2015). To verify possible genomic contamination, we used Kraken2 v2.0.8_beta with the database ftp://ftp.ccb.jhu.edu/pub/data/kraken2_dbs/minikraken_8GB_202003.tgz (Davis et al., 2013). The genomes’ assembly was performed with SPAdes v3.15.5 with k-mer sizes equal to 21, 33, 55, 77, 99, and 127 and the options error correction and carefully activated (Bankevich et al., 2012).

After assembly, the genomic sequences of each isolate were compared with sequences from species of *Mycobacterium* genus terrae-complex deposited in the GenBank database to determine the Average Nucleotide Identity (ANI) and the digital DNA-DNA hybridisation (dDDH) (Meier-Kolthoff et al., 2013). To determine ANI, the online tool *ANI calculator*^2^ was used (Konstantinidis and Tiedje, 2005; Goris et al., 2007; Yoon et al., 2017; Tortoli et al., 2019). To calculate dDDH, we used the tool *Genome-to-Genome Distance Calculator* (GGDC) v2.1,^3^ using the values provided by the formula 2 (identities/HSP length) (Tortoli et al., 2019). To classify genomes as belonging to a particular species, we used the cut-off ANI > 95% and/or dDDH > 70%, while for classification as a subspecies, the cut-off was ANI ≤ 97% together with dDDH ≤ 80% (Meier-Kolthoff et al., 2013; Tortoli et al., 2019).

### 2.6 Phylogenetic analysis

We used MEGA-Molecular Evolutionary Genetics Analysis v.11.0.13 software to construct the phylogenetic trees based on partial genes. The *hsp65*, *rpoB*, and *16S* rRNA gene sequences were first aligned with the CLUSTALW tool integrated into the MEGA program. The Neighbor-joining (N-J) statistical method and the Kimura 2-parameter nucleotide substitution model were used for the analyses. Bootstrap values were calculated from 1,000 replications to determine the branch significances. The support for each branch, derived from 1,000 bootstrap samples, is shown at the base of each node. The bar length (Bar 0.02) corresponds to two nucleotide substitutions per 100 nucleotides. Bootstrap values exceeding 50% were considered statistically significant.

A phylogenetic tree based on WGS was constructed that was based on, in addition to the genomes proposed as new species in the present study (MYC017, MYC098, MYC101, MYC123, and MYC340), 27 additional bacterial genomes representative of each clade of *Mycobacterium* genus (Supplementary Table 2). *Hoyosella altamirensis* was used as the outgroup, as described earlier (Tortoli et al., 2019).

All genomic sequences were annotated with Prokka v1.14.6 (employing the parameter “–kingdom Bacteria”) to standardize the protein prediction (Seemann, 2014). We used Ortho Finder v2.5.2 (Emms and Kelly, 2019) (applying the “-M msa” option) to extract the shared single copy orthologs and create the multiple alignments based on 935 genes using MAFFT v7.471 (Katoh et al., 2002). The resulting alignment was fed into RAxML-NG v1.0.1 (Kozlov et al., 2019) to generate a maximum-likelihood (ML) phylogeny, applying the “–model LG + G + F” with 500 bootstrap replicates. The tree was employed, and the midpoint was rooted in iTOL v5.0 (Letunic and Bork, 2021). The bioinformatics pipeline is described at https://doi.org/10.5281/zenodo.4660336.

### 2.7 Chemotaxonomy

Matrix-assisted laser Desorption/Ionization Time-of-Flight mass spectrometry (MALDI-TOF-MS) analysis was conducted as chemotaxonomy biological classification for the five mycobacterial isolates using a published protocol (El Khéchine et al., 2011). In addition, the reference strains *M. fortuitum* DSM46621 and *M. terrae* ATCC15755 (referred to as M.F. and M.T., respectively) were also subjected to MALDI-TOF-MS analysis. After extracting proteins, triplicate samples of 1 μL each were placed in a 96-well desorption plate (MSP; Bruker Daltonics). The samples were then covered with 1 μL of α-cyano-4-hydroxycinnamic acid matrix dissolved in a solution of 50% acetonitrile and 2.5% trifluoroacetic acid, allowing the samples to crystallize for analysis. A MALDI-TOF LT Microflex Bruker mass spectrometer was utilized with a 337 nm nitrogen laser in linear mode, controlled by the Flex Control v3.4 computational program (Bruker Daltonics, Billerica, United States).

The resulting spectra were saved using a laser frequency of 60HzA, with a voltage of 20 kV for ion source 1, 18.6 kV for ion source 2, and 9.28 kV for the lens. The mass range of analysis was set from 2,000 to 20,000 KDa. Multiple laser shots were performed automatically in different areas of each sample, resulting in the collection and analysis of 240 spectra. The equipment was calibrated using *Escherichia coli* DH5α extract. The generated spectra were analyzed with the BioTyper v10.0 (MBT) software (Bruker Daltonics, Billerica, United States). This software compares the obtained spectra from each sample with the profiles available in the database and generates a score value from 0 to 3. The score ranges from 0.000 to 1.699 when the sample is not identified, from 1.700 to 1.999 when there is a probable identification of the genus, from 2.000 to 2.299, indicating a secure identification of the genus and potential species, and finally from 2.300 to 3.000 representing a secure identification of the species.

## 3 Results

**FIGURE 1:**
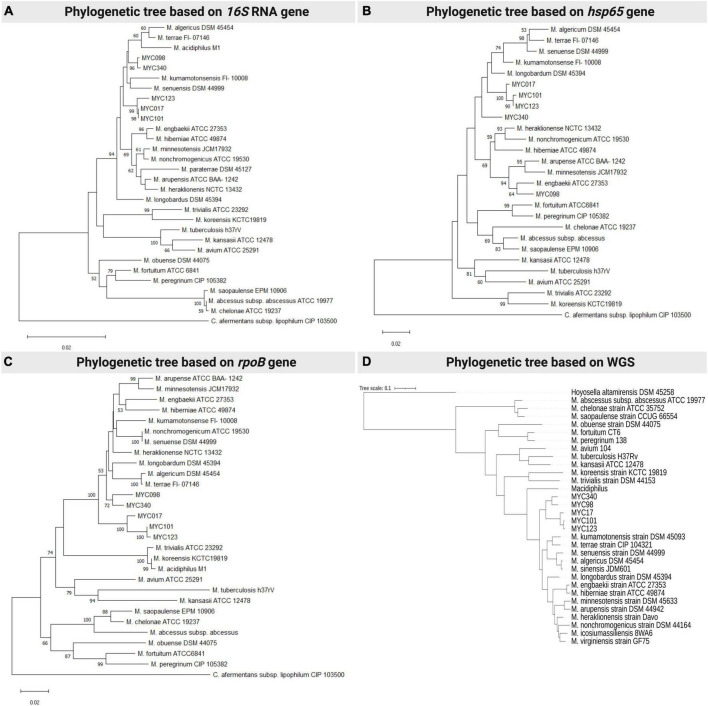
Phylogenetic analysis of representative species of the *Mycobacterium terrae* complex and the MYC strains (17, 98, 101, 12, 340), using neighbour-joining statistical methods and the Kimura 2-parameter nucleotide substitution model methods, with the following targets/genes/conserved regions: **(A)** 16S rRNA gene; **(B)** hsp65 gene; and **(C)** rpoB gene. **(A,D)** comparative analysis was performed using complete genome sequencing data from the MYC strains and available genomes in Genbank. Each panel is a representative result among four experiments.

Initial characterization of the isolates was carried out by sequencing and analyzing the *hsp*65, *rpo*B and 16S rRNA genes in addition to phenotypic and biochemical characteristics. The phylogenetic trees clearly separated the newly proposed species from the others already described, uniting them in a clade closely related to MTC (Figures 1A–C, respectively). Furthermore, it is shown that MYC017 is very closely related to MYC101 and MYC123, while MYC098 is related to MYC340 by analysis of the *rpo*B and 16S rRNA and related to *Mycobacterium engbaekii* by the *hsp*65 gene. In addition, all isolates presented the insertion of two nucleotides in helix 18 of the 16S rRNA gene, in comparison with other slow-growing mycobacteria, as well as species of MTC (Supplementary Figure 1).

Microscopic examination of isolated colonies after ZN staining showed that all were acid-fast bacilli. All isolates grew in seven days at 30 and 37°C, and were positive for the semiquantitative catalase, TCH growth and nitrate reduction tests, but negative for the MacConkey growth tests, NaCl growth, iron uptake, and growth at 42°C. Variable results were observed for urease tests, Tween 80 hydrolysis and tellurite reduction, in which only the MYC340 isolate was positive for all these tests. Furthermore, none of the isolates produced pigment and were therefore classified as non-chromogenic. The phenotypic and biochemical tests for the new bacterial species are described in Table 1. Variable results from phenotypic tests are also presented by the species belonging to the MTC as well as by the isolates in this study (Supplementary Table 3).

**TABLE 1:** Phenotypic and biochemical results performed for five isolates proposed as new species.

	Isolate ID of the new proposed species				
Test name	MYC017^T^	MYC098^T^	MYC101^T^	MYC123	MYC340^T^
Growth in 7H10 (days)	7	7	7	7	7
Growth in LJ (days)	7	7	7	7	7
Growth in TCH	+	+	+	+	+
Growth in MacConkey	–	–	–	–	–
Growth in NaCl	–	–	–	–	–
Urease	–	+	–	–	+
Tween 80 Hydrolysis	–	+	+	+	+
Tellurite Reduction	+	–	+	+	+
Semiquantitative catalase	+	+	+	+	+
Iron Capture	–	–	–	–	–
Nitrate Reduction	+	+	+	+	+
Growth at 30°C	+	+	+	+	+
Growth at 37°C	+	+	+	+	+
Growth at 42°C	–	–	–	–	–
Pigmentation	–	–	–	–	–

(+) indicates a positive result and (−) indicates a negative result. TCH, 2-thiophenecarboxylic acid hydrazide; LJ, Löwenstein-Jensen medium.

The AST results based on MIC are described in Table 2 which shows that four isolates are susceptible to amikacin, ciprofloxacin, clarithromycin, sulfamethoxazole-trimethoprim, moxifloxacin, and resistant to rifampicin, isoniazid, streptomycin and ethambutol. Isolates MYC017, MYC101, and MYC123 are susceptible to doxycycline, while MYC340 is resistant.

**TABLE 2:** Antimicrobial susceptibility results based on Minimum Inhibitory Concentration (MIC) (μg/mL) for isolates in this study.

	Isolate identification of the new proposed species			
Antimicrobial	MYC017^T^	MYC101^T^	MYC123	MYC340^T^
AMK	≤1	≤1	≤1	4
CIP	≤0.125	≤0.125	≤0.125	≤0.125
CLA	≤0.5	≤0.5	≤0.5	≤0.5
DOX	≤0.25	≤0.25	≤0.25	8
SUT	≤0.25/4.75	≤0.25/4.75	≤0.25/4.75	1/19
MOX	2	≤0.25	≤0.25	≤0.25
RIF	>16	>16	>16	>16
INH	>32	>32	>32	>32
SPM	>32	>32	>32	>32
EMB	>32	>32	>32	>32

AMK, Amikacin; CIP, Ciprofloxacin; CLA, Clarithromycin; DOX, Doxycycline; SUT, Trimethoprim + Sulfamethoxazole; MOX, Moxifloxacin; RIF, Rifampicin; INH, Isoniazid; SPM, Streptomycin; EMB, Ethambutol. Isolate MYC098^T^ was not able to grow in Muller-Hinton media.

The genomes were sequenced, and assembly and annotation were performed (Table 3). Quality control based on taxonomic classification is presented in Supplementary Figure 2. The results of the comparison of ANI and dDDH among the five isolates and their comparison with species of MTC are shown in Supplementary Table 4. Isolates MYC101 and MYC123 presented values for ANI = 98.57 and dDDH = 87.30, therefore belonging to the same species. The other isolates presented values for ANI and dDDH below the cut-off point (<95% for ANI and <70% for dDDH) and, as such, represent different species. Comparative analysis of ANI and dDDH between the genomes of the five isolates in this study and the species of the MTC with available genomes showed values below the established cut-off point, thus configuring four new species of this genus.

**TABLE 3:** Sequencing, assembly and annotation results for five isolates proposed as new species of the *Mycobacterium terrae* complex.

	Genomes ID				
General features	MYC017^T^	MYC098^T^	MYC101^T^	MYC123	MYC340^T^
Genome size	4.862408	5.180050	4.834772	4.636646	5.666799
Paired-end reads	6.270.197	6.668.384	6.513.480	2.408.306	2.120.357
Contigs	91	58	87	86	208
N50 (Kb)	181.022	251.295	126.619	147.492	159.838
Completeness (%)	100	100	100	100	100
Contamination	0.15	1.24	0.15	0.15	3.31
G + C (%)	67.72	67.84	67.72	67.89	67.63
CDSs	4.626	4.854	4.657	4.457	5.400
rRNA operons	447	347	347	347	349
tRNAs operons	52	74	53	52	74
Coverage (X)	322	322	336	129	94

The ML phylogenetic tree showed that isolates of new species were grouped within the MTC, but in a specific subclade separated from the species already described and supported by 100% bootstrap value, confirming that they are part of the *Mycobacterium* genus within the terrae clade and different from already established species (Figure 1D).

**FIGURE 2:**
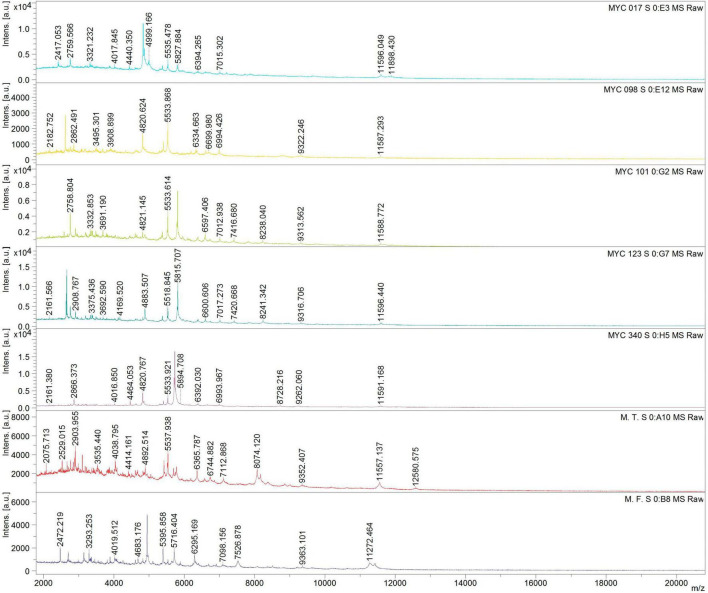
Comparative Matrix-assisted laser desorption/ionization time-of-flight mass spectrometry (MALDI-TOF-MS) of MYC isolates (17, 98, 101, 123, 340) and reference strains *Mycobacterium terrae* (M.T) and *Mycobacterium fortuitum* (M.F) identified different mass-to-charge (m/z) peak patterns, indicating potential novel species within the MYC group.

The five isolates and reference strains of *Mycobacterium fortuitum* and *M. terrae* were subjected to MALDI-TOF-MS analysis. The obtained spectra were then compared to those in the database using the Biotyper software, resulting in a value score for each. The reference strains, *M. fortuitum* and *M. terrae* were correctly identified with scores of 2.2 and 2.0, respectively, while the analyzed MYC isolates exhibited spectra of good quality but had identification scores ranging from 1.4 to 1.6, therefore not being identified (Figure 2). Additionally, it is noteworthy that MYC101 and MYC123, identified as the same species through genomic analyses, displayed indistinguishable profiles, confirming their similar nature.

Supplementary Table 5 details the correlation between culture isolates, species nomenclature, and genomic identifications from biorepositories.

## 4 Discussion

Description of novel Prokatiotic taxa might have important implications for human health and biotechnological implications. In this sense, studies are essential for advancing our knowledge of microbial diversity, which has implications for fields as diverse as medicine, ecology, industry and environmental sciences. In our previous study, a large sampling of mycobacteria from water samples from the São Paulo Zoological Park was evaluated and the results suggested the presence of a putative new species (Romagnoli et al., 2020). From these, five isolates were selected for further characterization regarding their taxonomic position.

Currently, the *Mycobacterium* genus is formed up of 196 species (number of child taxa with a validly published and correct name) (LPSN, 2023), the majority of which are found in natural environments such as lakes, rivers, swamps, and soil, as well as water treatment and distribution systems (Falkinham, 2015; Claeys and Robinson, 2018). Phenotypic, biochemical and molecular characteristics are useful for differentiating between species. Growth time is particularly useful for dividing species into three large groups: fast-growing (visible colonies within 7 days), slow-growing (growing of visible colonies after 7 days) and intermediate-growing (growing of visible colonies from 5 to 15 days) organisms. In this work, biochemical and cultural tests were not able to clearly distinguish the five isolates from other non-pigmented mycobacteria but could be classified by growth time as intermediate, characteristic compatible with species of the MTC.

The antimicrobial susceptibility profile presented by the isolates in this study was also observed in other species of the *M. terrae* complex (Hannigan et al., 2013; Tortoli et al., 2013; Djouadi et al., 2016). It is important to note that the susceptibility variability has also been reported for several isolates of *M. arupensis* (Cloud et al., 2006). Thus, it is not possible to state that the results observed in the isolates in this study represent the profile of each new species.

Analysis of three housekeeping genes showed that all isolates are related to the MTC and is according to the intermediate growth characteristic. In addition, the isolates have an insertion of two nucleotides in the helix 18 of the 16S rRNA gene. It is consense that this simple two-nucleotide insertion remains one of the most important features to determine whether a mycobacterium is related to the MTC (Tortoli et al., 2013; Abudaff and Beam, 2018).

For further characterization of these isolates, their genomes were sequenced for taxonomic analyses using ANI and dDDH. Genome sequences have gained extensive usage and pertinent software tools are easily accessible both as web services and as standalone applications. Furthermore, commonly adopted threshold values for ANI and dDDH to recognize species are 95–96 and 70%, respectively (Richter and Rosselló-Móra, 2009; Meier-Kolthoff et al., 2013; Chun et al., 2018). Our study showed values of ANI and dDDH between 81.59–85.56 and 24.4–28.8%, respectively, when compared to the genomes of species of the canonical *Mycobacterium* genus, confirming the classification of these isolates as new species.

Phylogenetic analysis used representative members from the five monophyletic groups, as previously suggested (Gupta et al., 2018), to construct the ML phylogenetic tree. The study was based on genomic sequences on molecular signatures identified in core proteins, dividing the genus *Mycobacterium* into five monophyletic groups, namely: *Mycolicibacterium* (Fortuitum-Vaccae clade), *Mycolicibacter* (Terrae clade), *Mycolicibacillus* (Triviale clade), *Mycobacteroides* (Chelonae-Abscessus clade) and *Mycobacterium* (Tuberculosis-Simiae clade). Thus, representative members from each of the five monophyletic groups were selected for analysis which showed that MYC isolates form a distinct cluster within the MTC (”Terrae Clade” or *Mycolicibacter*) and exhibit differences from previously established species. However, it is important to mention that the canonical genus *Mycobacterium* has been reconstituted and was therefore adopted here (Meehan et al., 2021).

Demonstrating whether a strain represents a new species or not can be adequately achieved with a genome sequence alone, without the obligatory generation of various types of chemotaxonomic data. Thus, for the proposal of new species, a comprehensive analysis of genome sequences, a basic assessment of growth characteristics, and the public deposition of type strains should be performed (Vandamme and Sutcliffe, 2021). We therefore have chosen this polyphasic analysis of characteristics such as morphology and growth conditions, physiology, genome and chemotaxonomy. In this context, it is worth highlighting that among the diverse chemotaxonomic information available, MALDI-TOF mass spectrometry analysis has demonstrated usefulness not only for routine diagnostic microbiology but also contributes to taxonomy as the detection of new protein profiles and can therefore readily suggest new species (Rodriguez-Temporal et al., 2017; Alcaide et al., 2018; López Medrano et al., 2021; Oviaño and Rodríguez-Sánchez, 2021; Li et al., 2022). The availability of freely accessible and continuously updated spectral databases would undoubtedly be of great benefit to the systematics community and would further promote the adoption of this technology (Vandamme and Sutcliffe, 2021). In this study, the MALDI-TOF results align with other data indicating that the MYC isolates represent previously unidentified species and also support that isolates MYC101 and MYC123 belong to the same species.

In conclusion, we present evidence to classify the strains isolated (MYC017, MYC098, MYC101, MYC123 and MYC340) from waste samples in a zoo in the state of São Paulo – Brazil as four new species of the genus *Mycobacterium*, considering MYC101 and MYC123 a single species.

## 5 Nomenclature

### 5.1 Description of *Mycobacterium vasticus* sp. nov

*Mycobacterium vasticus* (vas.ti.cus. L. masc. adj. vastum). Waste and sewage are characteristics of the origin of this isolate. Growth on solid Middlebrook 7H10-OADC medium and Löwenstein–Jensen medium requires 7 days at 30°C and 37°C, giving rise to shiny, smooth, non-chromogenic colonies with defined edges and creamy consistency. Growth in TCH was observed, neither in NaCl nor in MacConkey. *M. vasticus* is positive for catalase production and nitrate and tellurite reduction, while it is negative for Tween 80 hydrolysis, urease production and iron uptake. AST revealed susceptibility to amikacin, ciprofloxacin, clarithromycin, sulfamethoxazole-trimethoprim, moxifloxacin, and doxycycline, but resistance to ethambutol, rifampicin, isoniazid, and streptomycin. The type strain is MYC017^T^ (= ATCC TSD-296^T^ = JCM 35364^T^). The GenBank accession numbers for the draft genome, Biosample and 16S rRNA gene sequence are, respectively, CP084028, SAMN20959233 and MK890459.1.

### 5.2 Description of *Mycobacterium crassicus* sp. nov

*Mycobacterium crassicus* (cras.si.cus. L. masc. adjt. crassa). Crude sewage is characteristic of the environment from where the isolate came. Growth on solid Middlebrook 7H10-OADC and Löwenstein–Jensen medium requires 7 days at 30°C and 37°C, giving rise to shiny, smooth, non-chromogenic colonies with defined edges and creamy consistency. Growth in TCH, but not in NaCl or MacConkey was observed. *M. crassicus* is positive for catalase production, urease production, Tween 80 hydrolysis and nitrate reduction while negative for tellurite reduction and iron uptake. The type strain is MYC098^T^ (= ATCC TSD-297^T^ = JCM 35365^T^). The GenBank accession numbers for the draft genome, Biosample and 16S rRNA gene are, respectively: CP084029, SAMN20959234, and MK890478.1.

### 5.3 Description of *Mycobacterium zoologicum* sp. nov

*Mycobacterium zoologicum* (zoo.lo.gi.cum. L. masc. subst. zoo.) was isolated from a zoo’s sewage treatment plant. Growth in solid Middlebrook 7H10 medium and on Löwenstein–Jensen medium requires 7 days at 30°C and 37°C, giving rise to non-chromogenic, shiny, smooth colonies with defined edges and creamy consistency. The specie presents growth in TCH but not in NaCl or MacConkey. *M. zoologicum* is positive for Tween 80 hydrolysis, catalase production, nitrate, and tellurite reduction, while it is negative for urease production and iron uptake. They are susceptible to amikacin, ciprofloxacin, clarithromycin, sulfamethoxazole-trimethoprim, moxifloxacin, and doxycycline but resistant to ethambutol, rifampicin, isoniazid, and streptomycin. The type strain is MYC101^T^ (= ATCC TSD-298^T^ = JCM 35366^T^) and an additional isolate MYC123 (= ATCC BAA-3216 = JCM 35367). The GenBank accession numbers for the draft genome, Biosample and 16S rRNA gene sequence are, respectively: MYC101^T^ (CP084030, SAMN20959235, MK890479.1) and MYC123 (CP083985, SAMN20062777, MK890481.1).

### 5.4 Description of *Mycobacterium nativiensis* sp. nov

*Mycobacterium nativiensis* (na.ti.vi.en.sis. L. masc. subst. nativus.). From “native”, local where bacteria was isolated. Growth in solid Middlebrook 7H10 medium and on Löwenstein–Jensen medium was observed in 7 days at 30°C and 37°C, giving rise to non-chromogenic, shiny, smooth colonies with defined edges and creamy consistency. It shows growth in TCH but not in NaCl or MacConkey. *M. nativiensis* is positive for Tween 80 hydrolysis, catalase and urease production, and nitrate and tellurite reduction while negative for iron uptake. It is susceptible to amikacin, ciprofloxacin, clarithromycin, sulfamethoxazole-trimethoprim and moxifloxacin but resistant to ethambutol, rifampicin, isoniazid, doxycycline, and streptomycin. The type strain is MYC340^T^ (= ATCC TSD-299^T^ = JCM 35368^T^). The GenBank accession numbers for the draft genome, Biosample and 16S rRNA gene sequence are, respectively: CP083986, SAMN20062778 and MK890521.1.

## Data availability statement

The datasets presented in this study can be found in online repositories. The names of the repository/repositories and accession number(s) can be found in the article/Supplementary material.

## Author contributions

CLR: Conceptualization, Data curation, Formal analysis, Investigation, Methodology, Visualization, Writing – original draft, Writing – review and editing. ECC: Data curation, Formal analysis, Investigation, Methodology, Software, Visualization, Writing – original draft, Writing – review and editing. EM: Data curation, Formal analysis, Investigation, Methodology, Software, Visualization, Writing – original draft, Writing – review and editing. LB: Investigation, Methodology, Writing – review and editing. AS: Data curation, Formal analysis, Investigation, Methodology, Software, Visualization, Writing – original draft, Writing – review and editing. NS: Investigation, Methodology, Writing – review and editing. LM: Investigation, Methodology, Writing – review and editing. MJ: Investigation, Methodology, Writing – review and editing. ML: Data curation, Formal analysis, Investigation, Methodology, Writing – review and editing, Resources, Validation. LD: Writing – review and editing, Data curation, Formal analysis, Investigation, Methodology, Software. PS: Funding acquisition, Resources, Supervision, Writing – review and editing. SL: Conceptualization, Funding acquisition, Supervision, Writing – original draft, Writing – review and editing. CV-N: Conceptualization, Data curation, Formal analysis, Funding acquisition, Investigation, Methodology, Project administration, Resources, Software, Supervision, Visualization, Writing – original draft, Writing – review and editing.

## Footnotes

1
https://lpsn.dsmz.de/

2
https://www.ezbiocloud.net/tools/ani

3
http://ggdc.dsmz.de/ggdc.php
