# Correction: Description of new species of *Mycobacterium terrae* complex isolated from sewage at the São Paulo zoological park foundation in Brazil

**DOI:** 10.3389/fmicb.2026.1881582

**Published:** 2026-06-02

**Authors:** Camila Lopes Romagnoli, Emilyn Costa Conceiçao, Edson Machado, Leonardo Bruno Paz Ferreira Barreto, Abhinav Sharma, Natalia Maria Silva, Lucas Evangelista Marques, Maria Aparecida Juliano, Maria Cristina da Silva Lourenço, Luciano Antonio Digiampietri, Philip Noel Suffys, Sylvia Cardoso Leao, Cristina Viana-Niero

**Affiliations:** 1 Departamento de Microbiologia, Imunologia e Parasitologia, Universidade Federal de São Paulo, São Paulo, Brazil; 2 Laboratório de Bacteriologia e Bioensaios em Micobactérias, Instituto Nacional de Infectologia Evandro Chagas, Fundação Oswaldo Cruz, Rio de Janeiro, Brazil; 3 DSI-NRF Centre of Excellence for Biomedical Tuberculosis Research, SAMRC Centre for Tuberculosis Research, Division of Molecular Biology and Human Genetics, Faculty of Medicine and Health Sciences, Stellenbosch University, Cape Town, South Africa; 4 Laboratório de Biologia Molecular Aplicada a Micobactérias, Instituto Oswaldo Cruz, Fundação Oswaldo Cruz, Rio de Janeiro, Brazil; 5 Departamento de Biofísica, Universidade Federal de São Paulo, São Paulo, Brazil; 6 Escola de Artes, Ciências e Humanidades, Universidade Federal de São Paulo, São Paulo, Brazil

**Keywords:** *Mycolicibacter*, taxonomy, non-tuberculous mycobacteria, whole-genome sequencing, MALDI-TOF-MS, *Mycobacterium terrae* complex, Brazil

There was a mistake in Table 3, row nine, “rRNA operons”, as published. The following values in this row were given: “447, 347, 347, 347, 349”. The correct values are as follows: 4, 3, 3, 3, 3”. The corrected Table 3 appears below.

**Table 3:** Sequencing, assembly and annotation results for five isolates proposed as new species of the *Mycobacterium terrae* complex.

General features	Genomes ID				
	MYC017^T^	MYC098^T^	MYC101^T^	MYC123	MYC340^T^
Genome size	4.862408	5.180050	4.834772	4.636646	5.666799
Paired-end reads	6.270.197	6.668.384	6.513.480	2.408.306	2.120.357
Contigs	91	58	87	86	208
N50 (Kb)	181.022	251.295	126.619	147.492	159.838
Completeness (%)	100	100	100	100	100
Contamination	0.15	1.24	0.15	0.15	3.31
G+C (%)	67.72	67.84	67.72	67.89	67.63
CDSs	4.626	4.854	4.657	4.457	5.400
rRNA operons	4	3	3	3	3
tRNAs operons	52	74	53	52	74
Coverage (X)	322	322	336	129	94

In the abstract, “*Mycobacterium defluvii*” was mistakenly written instead of “*Mycobacterium vasticus”*. The corrected final sentence of the abstract is as follows: “According to these data, we propose the description of four new species belonging

to the *Mycobacterium* genus: (i) *Mycobacterium vasticus* sp. nov. strain MYC017^*T*^ (= ATCC TSD-296^*T*^ = JCM 35364^*T*^), (ii) *Mycobacterium crassicus* sp. nov. strain MYC098^*T*^ (= ATCC TSD-297^*T*^ = JCM 35365T), (iii) *Mycobacterium zoologicum* sp. nov. strain MYC101^*T*^ (= ATCC TSD-298^*T*^ = JCM 35366^*T*^) and MYC123 (= ATCC BAA-3216 = JCM 35367); and (iv) *Mycobacterium nativiensis* sp. nov. strain MYC340^*T*^ (= ATCC TSD-299^*T*^ = JCM 35368^*T*^).

