# Supplementary material for: Description of new species of Mycobacterium terrae complex isolated from sewage at the São Paulo zoological park foundation in Brazil
Source: Front Microbiol. 2024 Jan 23;15:1335985. doi: 10.3389/fmicb.2024.1335985 (PMC10844392; doi:10.3389/fmicb.2024.1335985)
Supplement: Supplementary file 1 [file Data_Sheet_1.zip › Supplementary Material/Supplementary table 3.docx]

| **Supplementary table 3.** Phenotypic and biochemical results of five isolates proposed as new species and from the *Mycobacterium terrae* complex. | | | | | | | | | | | |  |  |  |  |  |  |  |
| --- | --- | --- | --- | --- | --- | --- | --- | --- | --- | --- | --- | --- | --- | --- | --- | --- | --- | --- |
| **Characteristic** | **MYC017 ^T^** | **MYC098 ^T^** | **MYC101 ^T^** | **MYC123** | **MYC340 ^T^** | ***M. terrae* ATCC 15755****^T^** | ***M. nonchromogenicum* ATCC19530^T^** | ***M. kumamotonenis* JCM 13453^T^** | ***M. algericus* DSM 45454^T^** | ***M. arupensis* DSM 44942^T^** | ***M. engbaekii* ATCC 27353^T^** | ***M. heraklionensis* NCTC 13432^T^** | ***M. paraterrae* DSM 45127^T^** | ***M. hiberniae* ATCC 49874T** | ***M. longobardus* DSM 45394^T^** | ***M. acidiphillus* KCTC 49392^T^** | ***M. minnesotensis* DSM 45633^T^** | ***M. senuensis* KCTC 19147^T^** |
| Growth in TCH | + | + | + | + | + | **+** | **+** |  |  | **+** |  |  | **+** |  |  |  |  | + |
| Growth in MacConkey | - | - | - | - | - | **-** | **-** |  | **-** | **-** | **-** | **-** | **-** |  | **+** |  | **-** | - |
| Growth in NaCl | - | - | - | - | - | **-** | **-** | **-** | **-** | **-** |  |  | **-** | **-** |  | **+** | **+** | - |
| Urease | - | + | - | - | + | **-** | **-** | **-** | **+** | **-** | **-** | **-** | **-** | **-** | **+** | **-** | **-** | - |
| Tween 80 Hydrolysis | - | + | + | + | + | **+** | **+** | **+** |  | **+** | **+** | **-** | **-** | **+** | **+** | **-** | **-** | + |
| Tellurite Reduction | + | - | + | + | + | **-** | **+** |  | **+** |  | **+** | **-** | **-** |  | **-** |  |  | + |
| Semiquantitative catalase | + | + | + | + | + |  |  |  | **+** | **+** | **+** | **+** |  | **+** | **+** |  |  |  |
| Iron Capture | - | - | - | - | - |  |  |  |  | **+** |  |  |  |  |  |  |  |  |
| Nitrate Reduction | + | + | + | + | + | **+** | **+** | **+** | **+** | **-** | **-** | **+** | **+** | **+** | **+** | **+** | **-** | + |
| Growth at 30°C | + | + | + | + | + |  |  |  |  | **+** |  |  |  |  |  | **+** | **+** |  |
| Growth at 37°C | + | + | + | + | + | **+** | **+** |  | **+** |  |  |  | **+** | **+** |  | **+** | **-** | + |
| Growth at 42°C | - | - | - | - | - | **-** |  |  |  | **-** |  |  |  |  |  |  |  |  |
| Pigmentation | - | - | - | - | - | **-** | **-** | **-** | **-** | **-** | **+** | **-** | **-** | **+** | **-** | **-** | **+** |  |
