# Supplementary material for: Description of new species of Mycobacterium terrae complex isolated from sewage at the São Paulo zoological park foundation in Brazil
Source: Front Microbiol. 2024 Jan 23;15:1335985. doi: 10.3389/fmicb.2024.1335985 (PMC10844392; doi:10.3389/fmicb.2024.1335985)
Supplement: Supplementary file 1 [file Data_Sheet_1.zip › Supplementary Material/Supplementary table 4.docx]

**Supplementary table 4.** Results based on ANI and dDDH of the five new proposed species in comparison to the current species of the *Mycobacterium terrae* complex.

| Taxa A | Taxa B | ANI (%) | dDDH (%) |
| --- | --- | --- | --- |
| MYC017 | MYC098 | 86.56 | 31.7 |
| MYC017 | MYC101 | 94.65 | 58.5 |
| MYC017 | MYC123 | 94.23 | 56.1 |
| MYC017 | MYC340 | 86.18 | 31.2 |
| MYC098 | MYC101 | 86.15 | 30.7 |
| MYC098 | MYC123 | 85.98 | 30.6 |
| MYC098 | MYC340 | 92.29 | 46 |
| MYC101 | MYC123 | 98.57 | 87.3 |
| MYC101 | MYC340 | 86.6 | 31 |
| MYC123 | MYC340 | 85.96 | 30.8 |
|  |  |  |  |
| MYC017 | *M. algericus* DSM 45454 | 83.44 | 26.5 |
| MYC017 | *M. arupensis* DSM 44942 | 82.01 | 24.7 |
| MYC017 | *M. engbaekii* ATCC 27353 | 82.34 | 25.1 |
| MYC017 | *M. heraklionensis* 1245976.6 | 83.13 | 26.1 |
| MYC017 | *M. hiberniae* ATCC 49874 | 82.11 | 24.8 |
| MYC017 | *M. icosiumassiliensis* 8WA6 | 83.55 | 26.5 |
| MYC017 | *M. kumamotonensis* DSM 45093 | 83.75 | 27 |
| MYC017 | *M. longobardum* DSM 45394 | 83.7 | 27.3 |
| MYC017 | *M. minnesotensis* DSM 45633 | 81.85 | 38 |
| MYC017 | *M. nonchromogenicus* DSM 44164 | 82.51 | 42.6 |
| MYC017 | *M. senuensis* DSM 44999 | 83.53 | 26.4 |
| MYC017 | *M. sinensis* JDM601 | 83.1 | 26.3 |
| MYC017 | *M. terrae* NCTC 10856 | 84.07 | 27.3 |
| MYC017 | *M. virginiensis* GF75 | 83.1 | 26.2 |
|  |  |  |  |
| MYC098 | *M. algericus* DSM 45454 | 84.24 | 27.7 |
| MYC098 | *M. arupensis* DSM 44942 | 82.55 | 25.3 |
| MYC098 | *M. engbaekii* ATCC 27353 | 82.93 | 25.7 |
| MYC098 | *M. heraklionensis* 1245976.6 | 84.2 | 27.6 |
| MYC098 | *M. hiberniae* ATCC 49874 | 82.92 | 25.6 |
| MYC098 | *M. icosiumassiliensis* 8WA6 | 84.47 | 27.8 |
| MYC098 | *M. kumamotonensis* DSM 45093 | 84.73 | 28.3 |
| MYC098 | *M. longobardum* DSM 45394 | 84.47 | 28.3 |
| MYC098 | *M. minnesotensis* DSM 45633 | 82.45 | 25.1 |
| MYC098 | *M. nonchromogenicus* DSM 44164 | 83.37 | 26.3 |
| MYC098 | *M. senuensis*DSM 44999 | 84.27 | 27.6 |
| MYC098 | *M. sinensis* JDM601 | 84.08 | 27.5 |
| MYC098 | *M. terrae* NCTC 10856 | 84.78 | 28.4 |
| MYC098 | *M. virginiensis* GF75 | 84.08 | 27.3 |
|  |  |  |  |
| MYC101 | *M. algericus* DSM 45454 | 83.39 | 26.6 |
| MYC101 | *M. arupensis* DSM 44942 | 82.14 | 25.1 |
| MYC101 | *M. engbaekii* ATCC 27353 | 82.26 | 25.1 |
| MYC101 | *M. heraklionensis* 1245976.6 | 83.11 | 26.2 |
| MYC101 | *M. hiberniae* ATCC 49874 | 82.03 | 24.8 |
| MYC101 | *M. icosiumassiliensis* 8WA6 | 83.5 | 26.6 |
| MYC101 | *M. kumamotonensis* DSM 45093 | 83.68 | 26.8 |
| MYC101 | *M. longobardum* DSM 45394 | 83.97 | 27.5 |
| MYC101 | *M. minnesotensis* DSM 45633 | 81.8 | 24.5 |
| MYC101 | *M. nonchromogenicus* DSM 44164 | 82.52 | 25.3 |
| MYC101 | *M. senuensis* DSM 44999 | 83.48 | 26.6 |
| MYC101 | *M. sinensis* JDM601 | 83.47 | 26.5 |
| MYC101 | *M. terrae* NCTC 10856 | 83.74 | 26.9 |
| MYC101 | *M. virginiensis* GF75 | 83.47 | 26.4 |
|  |  |  |  |
| MYC123 | *M. algericus* DSM 45454 | 83.6 | 26.5 |
| MYC123 | *M. arupensis* DSM 44942 | 82.18 | 24.9 |
| MYC123 | *M. engbaekii* ATCC 27353 | 82.32 | 25 |
| MYC123 | *M. heraklionensis* 1245976.6 | 83.1 | 26.2 |
| MYC123 | *M. hiberniae* ATCC 49874 | 82.03 | 24.8 |
| MYC123 | *M. icosiumassiliensis* 8WA6 | 83.24 | 26.5 |
| MYC123 | *M. kumamotonensis* DSM 45093 | 83.7 | 26.8 |
| MYC123 | *M. longobardum* DSM 45394 | 84.17 | 27.8 |
| MYC123 | *M. minnesotensis* DSM 45633 | 81.59 | 24.4 |
| MYC123 | *M. nonchromogenicus* DSM 44164 | 82.54 | 25.4 |
| MYC123 | *M. senuensis*DSM 44999 | 83.55 | 26.6 |
| MYC123 | *M. sinensis* JDM601 | 83.4 | 26.4 |
| MYC123 | *M. terrae* NCTC 10856 | 83.71 | 26.9 |
| MYC123 | *M. virginiensis* GF75 | 83.34 | 26.7 |
|  |  |  |  |
| MYC340 | *M. algericus* DSM 45454 | 84.52 | 27.9 |
| MYC340 | *M. arupensis* DSM 44942 | 82.66 | 25.5 |
| MYC340 | *M. engbaekii* ATCC 27353 | 83 | 25.9 |
| MYC340 | *M. heraklionensis* 1245976.6 | 84.42 | 27.8 |
| MYC340 | *M. hiberniae* ATCC 49874 | 82.74 | 25.8 |
| MYC340 | *M. icosiumassiliensis* 8WA6 | 85.4 | 26.5 |
| MYC340 | *M. kumamotonensis* DSM 45093 | 84.91 | 28.7 |
| MYC340 | *M. longobardum* DSM 45394 | 84.79 | 28.6 |
| MYC340 | *M. minnesotensis* DSM 45633 | 82.57 | 25.4 |
| MYC340 | *M. nonchromogenicus* DSM 44164 | 83.6 | 26.5 |
| MYC340 | *M. senuensis* DSM 44999 | 84.43 | 27.8 |
| MYC340 | *M. sinensis* JDM601 | 84.32 | 27.9 |
| MYC340 | *M. terrae* NCTC 10856 | 85 | 28.8 |
| MYC340 | *M. virginiensis* GF75 | 85.56 | 26.7 |
