# Supplementary material for: Description of new species of Mycobacterium terrae complex isolated from sewage at the São Paulo zoological park foundation in Brazil
Source: Front Microbiol. 2024 Jan 23;15:1335985. doi: 10.3389/fmicb.2024.1335985 (PMC10844392; doi:10.3389/fmicb.2024.1335985)
Supplement: Supplementary file 1 [file Data_Sheet_1.zip › Supplementary Material/Supplementary table 1.docx]

**Supplementary Table 1.** Minimum inhibitory concentration (MIC) results for the newly proposed species proposed in this study (MYC017, MYC101, MYC123 and MYC340) and species of *M. terrae* complex

| **Drug** | **MYC017** | **MYC101** | **MYC123** | **MYC340** | ***M. engbaekii*** | ***M. heraklionense*** | ***M. longobardus*** | ***M. kumamotonense*** | ***M. icosiumassiliense*** | ***M. arupense*** | ***M. algericum*** | ***M. virginiense*** | ***M. senuense*** | ***M. sinense*** |
| --- | --- | --- | --- | --- | --- | --- | --- | --- | --- | --- | --- | --- | --- | --- |
| **AMK** | ≤1 | ≤1 | ≤1 | 4 | 2–8 | 1–32 | 32 | <0,5 | >64 | 16/32/64/128 | 1/10 | >8 |  | 32 |
| **CIP** | ≤0,125 | ≤0,125 | ≤0,125 | ≤0,125 | 2 | ≥16 | 16 |  | >32 | >32 |  | ≥16 |  | 15 |
| **CLAR** | ≤0,5 | ≤0,5 | ≤0,5 | ≤0,5 | 1,00 | 1 | 2 | 0,06 | >32 | 1/64 | 4/16/32/64 | 1 |  |  |
| **SUT** | ≤0,25/4,75 | ≤0,25/4,75 | ≤0,25/4,75 | 1/19 |  |  |  |  | >8/52 | ≥8/152- 4/76- 2/38- 1/19 |  | 1/19 |  |  |
| **MOX** | 2 | ≤0,25 | ≤0,25 | ≤0,25 | 2–4 | >8 | ≥8 |  | >8 | ≥16 | 05/2.5/10 | >8 |  |  |
| **DOX** | ≤0,25 | ≤0,25 | ≤0,25 | 8 | 16 | ≥16 | 4 |  | >16 |  |  | >16 |  |  |
| **EMB** | >32 | >32 | >32 | >32 | ≤0.5 | 2 | 4 | 0,5 | >16 | 0,5- 1 | 5/50 | 4 | 2 | >0,5 |
| **RIF** | >16 | >16 | >16 | >16 | 4 | 1/32 | 32 | 0,06 | 8 | 8/≥16 | 1/10 |  | 40 |  |
| **INH** | >32 | >32 | >32 | >32 |  |  |  | >32 | >16 |  |  |  | 0,2 | >50 |
| **SPM** | >32 | >32 | >32 | >32 | 2–8 | 2– >64 | 32 | 1 |  | 32/≥64 |  |  | 10 | 32 |
| **CEF** | ≤2 | ≤2 | ≤2 | ≤2 |  |  |  |  |  |  |  |  |  |  |
| **TOB** | ≤0,25 | ≤0,25 | ≤0,25 | 16 |  |  |  |  | 32 |  |  |  |  |  |
| **TIG** | >16 | ≤0,125 | >16 | >16 |  |  |  |  |  |  |  |  |  |  |
| **Reference** | This study | This study | This study | This study | Tortoli et al., 2013 | Tortoli et al., 2013 | Tortoli et al., 2013 | Masaki et al., 2006 | Djouadi et al., 2016 | Cloud et al., 2006 | Sahraoui, 2011 | Vasireddy,2016 | Ho-Suk Mun, 2008 | Zhang 2013 |
