# Supplementary figures and images for: Description of new species of Mycobacterium terrae complex isolated from sewage at the São Paulo zoological park foundation in Brazil
Source: Front Microbiol. 2024 Jan 23;15:1335985. doi: 10.3389/fmicb.2024.1335985 (PMC10844392; doi:10.3389/fmicb.2024.1335985)

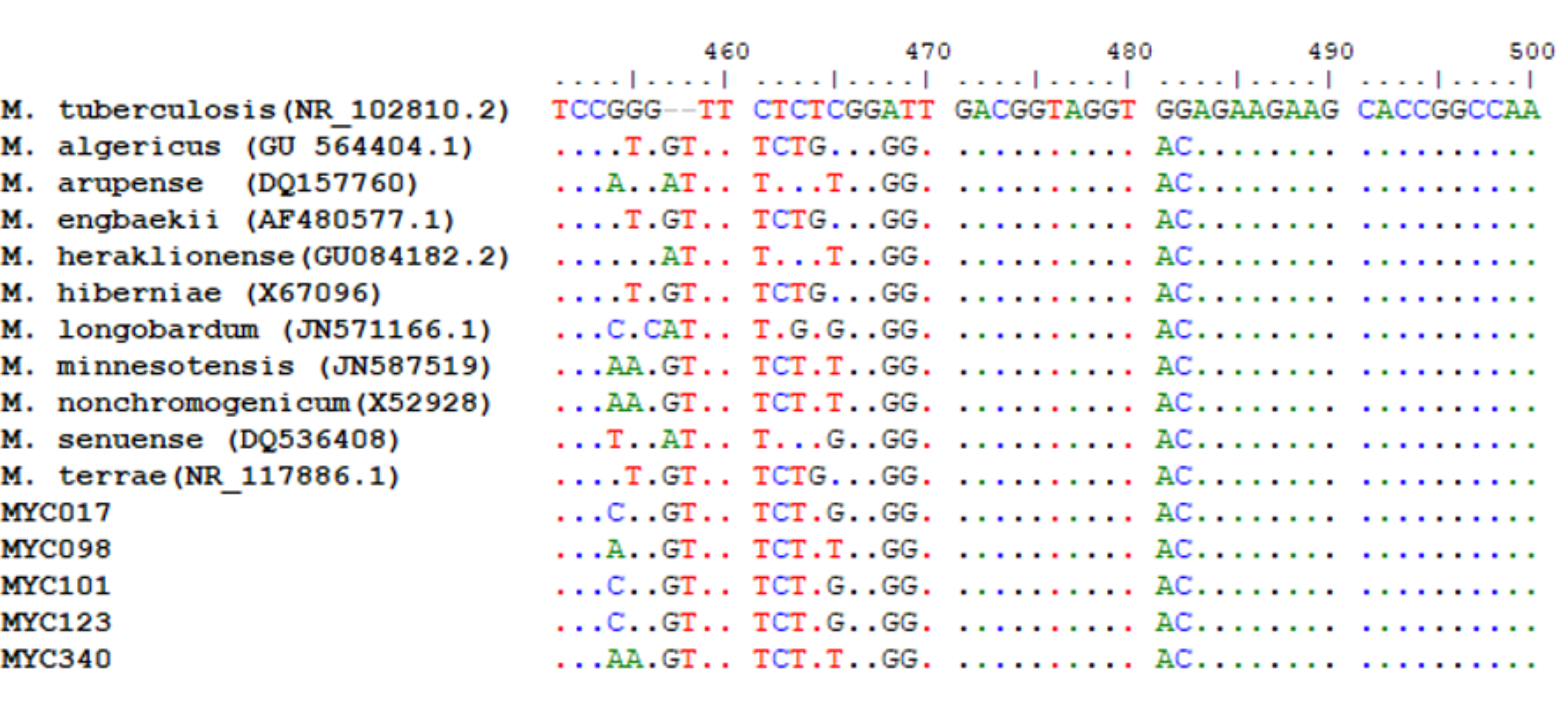

Supplement: Supplementary file 1 [file Data_Sheet_1.zip › Supplementary Material/Supplementary figure 1.png]
